# *Actinomadura meyerae *osteitis following wound contamination with hay in a woman in France: a case report

**DOI:** 10.1186/1752-1947-5-32

**Published:** 2011-01-26

**Authors:** Emilie Bonnet, Xavier Flecher, Sebastien Paratte, Jean-Noël Argenson, Didier Raoult, Pierre-Edouard Fournier

**Affiliations:** 1 Federation de Microbiologie, Hôpital de la Timone, Marseille, France; 2 Service de Chirurgie Orthopédique, Hôpital Sainte-Marguerite, Marseille, France

## Abstract

**Introduction:**

Mycetoma is a chronic granulomatous infection caused by environmental fungi or bacteria. It affects dermal and subcutaneous tissues, with putative contiguous extension to muscles or bones. While common in tropical and subtropical areas, mycetoma is rare in Europe.

**Case presentation:**

We describe a case of *Actinomadura meyerae *osteitis in a 49-year-old Caucasian woman who suffered a tibia open fracture contaminated with hay; to the best of our knowledge the first case of autochthonous *A. meyerae *infection reported in France. The bacterium was cultivated from a bone biopsy. Following surgical osteosynthesis and six months of treatment with cotrimoxazole, our patient made a full recovery.

**Conclusion:**

Our case report suggests that *A. meyerae *is a potential agent of wound infection in farm workers in contact with hay.

## Introduction

Infections caused by *Actinomadura *species, Gram-positive bacilli that belong to the family *Thermomonosporaceae *within the order *Actinomycetales*, are common in tropical and subtropical areas [1]. They are mostly caused by *Actinomadura madurae *and *Actinomadura pelletieri*. In Europe, only four cases of *Actinomadura *infections have been reported to date, caused by *Actinomadura *sp. [2,3], *A. madurae *[4] and *Actinomadura sputi *[5]. We report the first case of *Actinomadura meyerae *infection in a patient who developed osteitis following contamination of an open fracture wound with hay.

## Case presentation

**Figure 1:**
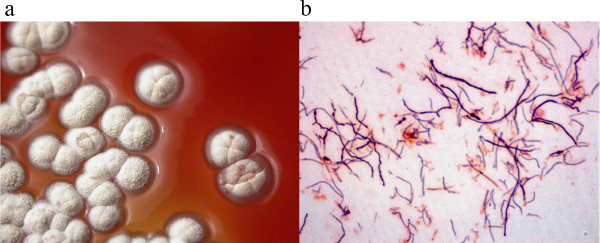
***Actinomadura meyerae *colonies**. A) *A meyerae *cultivated from our patient's bone biopsy on blood agar; B) Gram staining of an *A. meyerae *colony.

**Figure 2:**
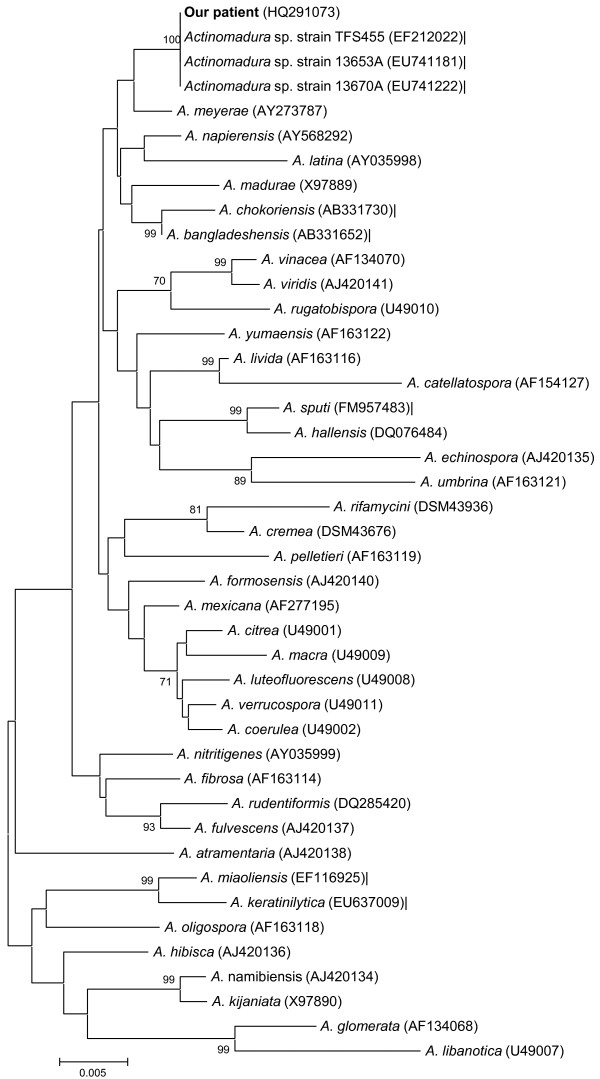
**Phylogenetic classification of the *Actinomadura meyerae *strain isolated from our patient's bone biopsy**. Unrooted dendrogram showing the phylogenetic classification of validated *Actinomadura *species. The analysis was based on 16S rRNA sequence comparison using the neighbor-joining method and MEGA software [7]. Bootstrap values greater than 70% are indicated at the nodes. The scale bar indicates a 0.2% sequence dissimilarity. GenBank accession numbers are indicated in parentheses.

A 49-year-old Caucasian woman working on a farm in Vichy, France, experienced an open fracture of the left tibia caused by a hook lifting a haystack. Subsequently, she was admitted to hospital. The fracture was reduced with a locking compression plate (Synthes, Oberdorf, Switzerland). Then, five days later, our patient developed a fever of 38.5°C, and oral treatment with amoxicillin-clavulanic acid, 6 g/day, was started; eight days after starting this treatment, the first antibiotic was replaced with oral ciprofloxacin (1.0 g/day) for three months. During that time, the cutaneous wound healed, but successive X-rays showed that bone healing was not obtained. Seven months following surgery, our patient broke her tibia locking compression plate while walking. Then, four weeks later, she was admitted to hospital. On examination, our patient had swelling and pain of the leg at the fracture site without any evident clinical sign of infection. X-rays showed a non-union of the tibia at the fracture site and the broken locking compression plate. Her blood test results showed a leukocyte count of 7.39 × 10^9 ^cells/L (73% polymorphonuclear cells, 24.9% mononuclear cells), a C reactive protein level of 1 mg/mL, and an erythrocyte sedimentation rate of 5 mm/first hour. The patient underwent removal of the locking compression plate, immediately followed by an intra-medullary nailing associated with a cancellous autologous bone graft harvested on the homolateral iliac crest. A peri-operative bone biopsy of the fracture site was performed in sterile conditions and sent to the microbiology laboratory of the Timone Hospital, Marseille, France. Direct microscopic analysis showed the presence of numerous polymorphonuclear leukocytes, but no bacteria were detected using Gram stain. The biopsy was cultivated in aerobic and anaerobic conditions at 37°C for one month. Plates were checked for bacterial growth twice a week. On the 28th day of culture, white colonies that were convex, powdery and adherent were detected on blood-enriched Columbia agar (Figure 1A). The strain was named Timone, according to the name of our hospital. Gram staining demonstrated that the colonies were made of irregular and extensively branched Gram-positive bacilli less than 1 μm in diameter (Figure 1B). The bacterium could not be stained using the Ziehl-Neelsen method. Its identification was obtained using 16S rRNA amplification and sequencing. Briefly, DNA was extracted from a colony using the QIAamp Tissue kit (Qiagen, Hilden, Germany) following the manufacturer's instructions. Polymerase chain reaction (PCR) amplification of a 1441 bp section was performed using the fD1 and rp2 primers as previously described [6]. Following comparison to sequences in the GenBank database (http://www.ncbi.nlm.nih.gov/genbank/; sequence deposited under accession number GenBank:HQ291073), the nucleotide sequence obtained from the PCR product was 100% identical to unpublished sequences recorded under accession numbers GenBank:EU741181, GenBank:EU741222, and GenBank:EF212022. The first two sequences were obtained from *Actinomadura *sp. from seawater from Costa Rica, and the latter from an *Actinomadura *sp. detected in shallow water sediments from Norway. By comparison with those of all officially validated *Actinomadura *species http://www.bacterio.cict.fr/index.html, our sequence exhibited a similarity ranging from 95.63% with *Actinomadura libanotica *(GenBank:AF163120) to 99.37% with *A. meyerae *(GenBank:AY273787). Using the MEGA software [7], we performed a phylogenetic analysis among *Actinomadura *species and found our patient's strain to be grouped with *A. meyerae *(Figure 2). The strain was deposited in the Collection de souches de l'Unité des Rickettsies (CSUR, WDCM875) under reference CSUR P15. The antimicrobial susceptibility of strain Timone was assessed using E-test strips (AB BIODISK, Solna, Sweden) on Mueller-Hinton agar. The strain was susceptible to gentamicin (minimal inhibitory concentration 0.5 μg/mL), doxycycline (0.25 μg/mL), cotrimoxazole (1 μg/mL) and vancomycin (2 μg/mL), and resistant to amoxicillin (8 μg/mL), imipenem (4 μg/mL), erythromycin (16 μg/mL) and rifampin (2 μg/mL). Subsequently, an oral treatment with cotrimoxazole (sulfamethoxazole, 4800 mg/day and trimethoprim, 960 mg/day) was started and continued for six months. Within 15 days of the onset of antibiotics, our patient's operative wound healed and local inflammatory symptoms resolved. A year later, our patient remains asymptomatic.

## Discussion

*Actinomadura *species are usually found in surface layers of the soil in semi-desertified areas of tropical and subtropical countries. Most human *Actinomadura *infections are caused by *A. madurae *and *A. pelletieri*. Both species are agents of mycetoma, a chronic and destructive cutaneous infection that may spread to the bones. Cases of *Actinomadura *infections have mostly been reported in India, the Middle East, Africa, and South America [1].

In Europe, *Actinomadura *infections have almost exclusively been diagnosed in immigrants from purported endemic areas [8], but rare endemic cases have also been reported. In Albania, a 45-year-old man with no history of travel to tropical or subtropical areas was diagnosed as having *Actinomadura *sp. infection of the foot [2]. In Greece, a 38-year-old man who reported no history of travel abroad presented with a chronic osteitis of the left foot caused by *A. madurae *[4]. A third autochthonous European case of *Actinomadura *sp. infection was described in Italy [3]. Recently *A. sputi *was isolated from the sputum of a German patient [5].

Our report is the fifth reported case of autochthonous *Actinomadura *infection in Europe, and the first in France. In addition, to the best of our knowledge, this is the first case of *A. meyerae *infection. As we isolated it in pure culture from a bone biopsy, we are confident that it was the causative agent of our patient's bone infection. *A. meyerae *was first described as *Actinomadura meyerii *in 2003 [9]. The type strain was isolated from a garden soil in Mexico City [9]. Our patient had not travelled to the usual endemic zones of mycetoma, but had a specific history of wound contamination with hay. This is the likely source of infection, as *Actinomadura *infection in mycetoma results from the introduction of this agent by minor percutaneous trauma, often associated with plant debris and/or soil. Using cotrimoxazole for six months due to the presence of internal fixation material, the infection improved rapidly, bone healing was obtained and our patient's infection was considered as cured one year later.

## Conclusion

Our report highlights the potential risk of *Actinomadura *infection in France, in particular in workers exposed to hay in agricultural settings, and identifies *A. meyerae *as a new pathogenic *Actinomadura *species.

## Consent

Written informed consent was obtained from the patient for publication of this case report and any accompanying images. A copy of the written consent is available for review by the Editor-in-Chief of this journal.

## Competing interests

The authors declare that they have no competing interests.

## Authors' contributions

EB and PEF wrote the manuscript while FF performed the bacteriological identification. XF and SP performed the surgical treatment and revised the manuscript for surgical content. JNA and DR corrected the manuscript. All authors read and approved the final manuscript.
